# Effects of Atmospheric Plasma Corona Discharge on *Saccharomyces cerevisiae:* Viability, Permeability, and Morphology

**DOI:** 10.3390/foods12020381

**Published:** 2023-01-13

**Authors:** Irina Amar Dubrovin, Efrat Emanuel, Yulia Lazra, Rivka Cahan

**Affiliations:** Department of Chemical Engineering, Ariel University, Ariel 40700, Israel

**Keywords:** atmospheric plasma corona discharge, yeasts, *Saccharomyces cerevisiae*, viability

## Abstract

Food spoilage is a routine challenge in food production. *Saccharomyces cerevisiae* is a major contaminating microorganism associated with fruit pulps and juices. Our study demonstrated the effect of a plasma corona discharge on *S. cerevisiae* viability, membrane permeability, and morphology when the cells were prepared in both dry and wet modes. The *S. cerevisiae* viability was examined as a function of the duration of plasma exposure, the sample’s distance from the treating head, initial cell concentration, and yeast suspension volume. The results showed a linear correlation between the exposure duration and the CFU/mL in both dry and wet modes. When the initial yeast concentration was 10^6^ CFU/mL, complete eradication in the dry and wet modes occurred after 45 and 240 s, respectively. Exposure of different initial concentrations of *S. cerevisiae* to plasma in dry (20 s) or wet (90 s) mode led to 2 to 3 orders of magnitude reduction. In both modes, there was total eradication when the initial cell concentration was about 10^3^ CFU/mL. The cell-membrane permeability was examined using a flow cytometer and the fluorescent dye propidium iodide (PI). Plasma treatment in the dry mode for 30 and 45 s led to 51% and 76% PI-positive cells. Similar results were obtained in the wet mode but with a longer exposure for 120 and 240 s, respectively. Atmospheric plasma may provide disinfection technology for the food industry in a short process without heating.

## 1. Introduction

Food spoilage is a common problem in the food industry. Factors that influence food spoilage are water availability and quality, storage temperature, pH, and initial microbial loads of fungi and bacteria [1]. Yeasts play an important role in the production of alcoholic and non-alcoholic beverages, the baking industry, and the production of volatile aroma compounds [2]. However, yeasts are commonly found in spoiling foods where bacterial growth is inhibited due to low pH and high sugar content [3]. *Saccharomyces cerevisiae* is a major contaminating microorganism associated with fruit pulps and packed fruit juices [4]. The inactivation of microorganisms in the food industry is commonly performed with thermal treatments [5]. For example, in the beverage industry, *S. cerevisiae* needs to be controlled at the end of the production process, and thermal treatment is mainly applied to prevent yeast proliferation. However, thermal treatment can affect nutritional and organoleptic properties [6]. Nonthermal disinfection methods include the addition of preservative compounds, such as the antimicrobial peptides Leg1 (RIKTVTSFDLPALRFLKL) and Leg2 (RIKTVTSFDLPALRWLKL) [7] or natural substances, such as phenolic compounds [8,9]. In several types of wine, such as sparkling wine, there is a final production step where the yeasts are removed by agitating and inclining the bottle, letting the yeasts settle into the bottleneck. However, this method takes two months [10,11]. Recently, physical nonthermal methods such as pulsed electric fields [12,13] and cold plasma have been used for microorganism eradication [14,15].

Atmospheric plasma corona discharge holds promise as an alternate technology, providing the advantage of a short process without heating. Plasma is an ionized gas containing an equal number of positively and negatively charged particles. It can be categorized as “hot” or “cold”. In hot plasma, the particles are in thermal equilibrium. Cold plasma is classified by its temperature corresponding to different particles, where the temperature of heavy particles is lower than the temperature of electrons (with an energy of 1–10 eV). The electrons’ energy is sufficient for the generation of energetic and chemically reactive species (excited atoms, free radicals, molecules, ions, and ultraviolet photons), which are the driving force for the plasma’s chemical reactions. Cold plasma can be generated under different gas pressures; low-pressure plasma discharges are created under a vacuum of 0.1–0.5 Torr. In contrast, atmospheric-pressure plasmas are generated under atmospheric pressure [16,17]. Cold-plasma technology is currently used in various fields, including surface modification [18], bioremediation of toxic pollutants using biofilm on plasma-pretreated wood waste [19], microbial electrolysis cells based on a pretreated plasma anode [20], advanced treatment of agricultural seeds [21], and water and soil remediation [17].

The plasma’s antimicrobial mechanism is believed to be based mainly on its reactive oxygen species (ROS) and reactive nitrogen species (RNS) [22]. They include hydroxyl hydrogen peroxide (H_2_O_2_), atomic oxygen (O), radicals (^•^OH), singlet oxygen (^1^O_2_), ozone (O_3_), [23,24], nitrates (NO_3_^−^), nitric oxide (NO), peroxinitrites (ONOO^−^), and nitrites (NO^2−^) [25]. These reactive molecules were found to alter the phospholipid bilayer of a cell, gene expressions and the structure of nucleic acids and cellular proteins [26,27].

Exposing *Listeria monocytogenes* grown on agar plates to an atmospheric-pressure plasma jet with He, He + O_2_, N_2_, or N_2_ + O_2_, for 2 min, led to cell reduction by 0.87, 4.19, 4.26, and 7.59 log units, respectively. When the *L. monocytogenes* were inoculated on sliced ham and chicken breast, the plasma treatment decreased the bacterial number by 1.94 to 6.52, and by 1.37 to 4.73 log, respectively. These results showed that the input gas used with the N_2_ + O_2_ mixture was the most efficient [28]. Braised chicken was air-packed and stored at 4 ± 1 °C for 15 days. During this period, the yeasts and molds increased sharply from 1.98 log (CFU/g) to 5.80 log (CFU/g). When the braised chicken was treated with dielectric barrier discharge cold plasma, molds and yeasts proliferation was significantly restrained [29]. Inactivation of *Zygosaccharomyces rouxii* LB and 1130 (initial concentration of about 1 × 10^7^ CFU/mL) in apple juice was studied using a gas-phase surface discharge plasma system at different peak discharge voltages. Plasma treatment for 30 min at a voltage of 11.3 kV, led to a reduction of *Z. rouxii* LB and 1130 by 2.39 and 2.60 log10. At a higher voltage of 21.3 kV, the reduction was 6.58 log10 and 6.82 log10, respectively. These results indicated that raising discharge voltage levels could decrease the plasma treatment time for microbial inactivation [30]. Recently, the effect of atmospheric plasma corona discharges on soil bacteria viability was examined. Exposure to the soil for 5 min led to a reduction of 2.5 orders of magnitude. The plasma-resistant bacteria were of the phylum Firmicutes (98.5%) and were comprised of the taxonomic orders *Bacillales* (95%) and *Clostridiales* (2%) [15]. Studying the effect of plasma corona on *Agrobacterium tumefaciens*, a soil-borne pathogenic bacterium, showed that, in a liquid environment, in an initial concentration of 2.02 × 10^6^ CFU/mL after 90 s of plasma exposure, there was a reduction of 5 orders of magnitude [14].

In our study, the effect of a plasma corona discharge on *S. cerevisiae* viability, membrane permeability, and morphology was examined, where the cells were prepared in both dry and wet modes. The *S. cerevisiae* viability was examined as a function of plasma exposure time, distance between the treating head and the sample, initial cell concentration, and the yeast suspension volume.

## 2. Materials and Methods

### 2.1. Plasma Corona Discharge System

**Figure 1:**
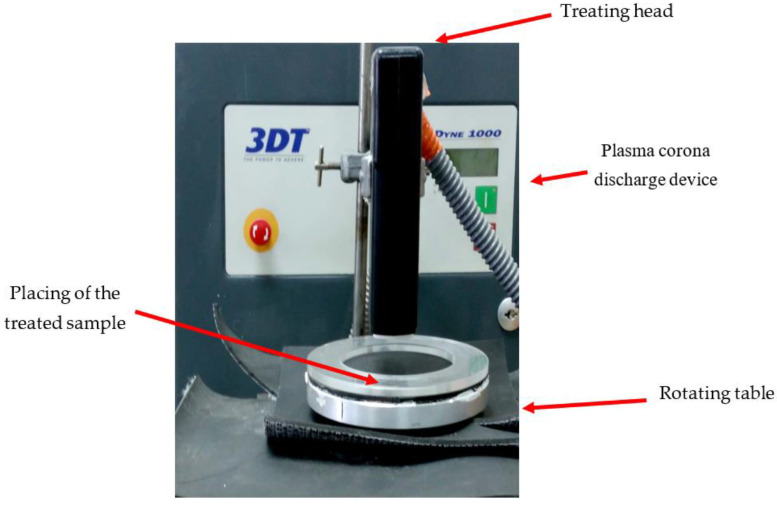
Plasma corona discharge device and the rotating table.

The plasma corona discharge device (3DT, MULTIDYNE 1000, Germantown, WI, USA) (Figure 1) used in this study was comprised of a treating head containing two electrodes. To generate the plasma, the device was operated under a high voltage of 2 × 12 kV and a frequency of 50 Hz under atmospheric pressure conditions, using ambient air as a carrier gas. A rotating table (Figure 1) covered with a layer of PVC was positioned beneath the treating head. The upper part of the table was rotated at controlled rounds per min (rpm) using a power supply (PowerPac^tm^ Basic, Bio-Rad, Hercules, CA, USA). A plastic Petri dish containing the *S. cerevisiae* sample was placed in the center of the rotating table.

### 2.2. Preparation of S. cerevisiae for Exposure to Plasma Corona Discharge, Experimental Conditions

*S. cerevisiae* (70468) was purchased from DSMZ (Braunschweig, Germany). It was grown in yeast mold broth (YMB) (Neogen, MI, USA) for 24–48 h at 30 °C and diluted to an appropriate OD (660 nm). The suspension was divided into 1 mL portions in Eppendorf tubes and washed in PBS as follows: centrifuged at 10,000 rpm for 10 min, decanted the supernatant, and suspended the *S. cerevisiae* sediment in 1 mL PBS. This final suspension of 10^3^–10^8^ CFU/mL was again centrifuged at 10,000 rpm for 10 min. When the experiment was conducted in dry mode, the *S. cerevisiae* sediment was suspended in 0.05 mL PBS and spread in the middle of a Petri dish (about 1 × 1 cm). For wet mode, the sediment was suspended in 10–30 mL PBS. The samples were exposed to plasma for 10–240 s, at a distance of 2–6 cm from the treating head, and rotated at 40 rpm as indicated for each experiment. The control samples were treated with the same procedure, excluding exposure to plasma.

### 2.3. Viable Count Assay

The live *S. cerevisiae* concentration was measured as a function of plasma treatment by a visible count assay. The plasma-treated dry-mode *S. cerevisiae* were harvested from the Petri dish with PBS (1 mL) into a sterile tube. A suspension (100 μL) was serially diluted, and the appropriate dilutions were pour-plated onto YM agar (Neogen, MI, USA), followed by incubation for 24–48 h at 30 °C. Viable *S. cerevisiae* cells were identified by CFU counting and multiplying it by the corresponding dilutions. The same procedure was carried out with the wet-mode *S. cerevisiae* cells, except that the 100 μL suspension was collected directly from the 10–30 mL in the Petri dish.

### 2.4. Examination of the S. cerevisiae Membrane Cell Size and Permeability by Flow Cytometry (FCM) Analysis

A volume of 1 mL of plasma-treated *S. cerevisiae* (10^6^ CFU/mL) and a nontreated sample were transferred to an Eppendorf tube, followed by the addition of fluorescent propidium iodide (PI) dye at a final concentration of 1.5 µM. The samples were incubated at 37 °C for 5 min. Afterward, 200 μL were transferred to an ELISA plate, and the *S. cerevisiae* sample (about 50,000 cells) was examined for membrane permeability using FCM (Beckman Coulter, Atlanta, GA, USA). Data were analyzed using FlowJo software (Tree Star, San Carlos, CA, USA).

### 2.5. Scanning Electron Microscope (SEM) Analysis

Plasma-treated *S. cerevisiae* and nontreated samples were washed gently (×3) with PBS. The *S. cerevisiae* (0.2 μL) were fixed by incubation in Karnovsky’s fixative solution (4% formaldehyde and 5% glutaraldehyde in 0.064 M phosphate buffer, pH 7.2), and were then incubated for 1 h in tannic acid (1%) and OsO4 (4%). After each process, the samples were washed three times with PBS (pH 7.2). Then the samples were dehydrated using ethanol (30–100%) and acetone (50–100%) for 10 min at each concentration. The samples were air-dried and sputtered with gold (using Quorum Q15OT ES, Quorum Technologies Ltd., Laughton, UK). The morphology of the *S. cerevisiae* cells was examined using a MAIA3 SEM (TESCAN, Kohoutovice, Czech Republic) at ultra-high resolution.

### 2.6. Statistics

Data were expressed as means ± STDEV (standard deviation) from 3 to 5 replicates.

## 3. Results and Discussion

### 3.1. S. cerevisiae Viability as a Function of Plasma Corona Discharge Exposure Duration

**Figure 2:**
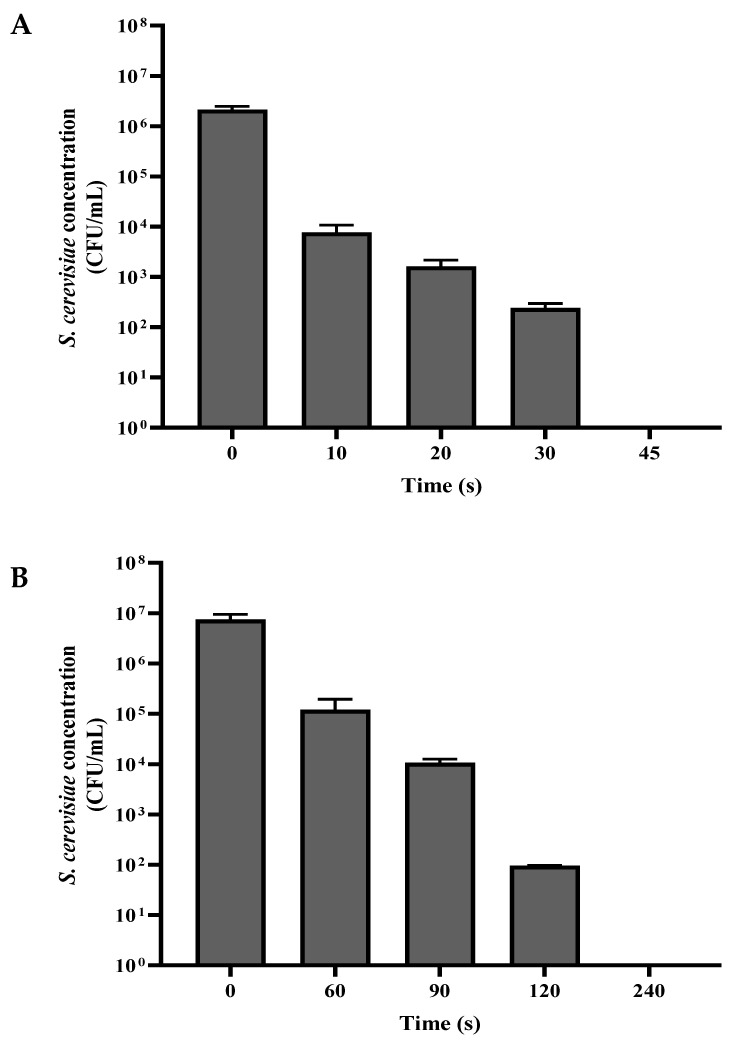
The concentration of the viable *S. cerevisiae* (CFU/mL) as a function of plasma corona exposure duration in dry mode (**A**) and wet mode (**B**).

A suspension of *S. cerevisiae* was washed with PBS, divided into volumes of 1 mL, and centrifuged. The washed *S. cerevisiae* sediment was exposed to a plasma corona discharge in dry mode (0.05 mL of the sediment dispersed on a Petri dish), and in wet mode (0.05 mL of the sediment diluted in 10 mL PBS and placed in a Petri dish). In both experiments, the Petri dish with the sample was placed under the plasma corona discharge treating head at a distance of 2 cm. The samples were exposed to plasma corona for 10–240 s in dry and wet mode. Following this process, the dry-mode samples were collected into an Eppendorf tube using 1 mL PBS. For wet mode, 1 mL of the 10 mL was collected for a viable count assay. The control samples received the same treatment but, without exposure to plasma corona. The concentration of viable *S. cerevisiae* as a function of plasma corona exposure duration is shown in Figure 2. In both modes, the first duration that is shown was a decrease in CFU/mL of about two orders of magnitude, and the last one is when total eradication was observed.

As shown in Figure 2A, when the *S. cerevisiae* cells were exposed to plasma corona, there was a linear correlation between the exposure duration and the CFU/mL in both dry mode and wet mode. When the experiment was performed in dry mode, the nontreated *S. cerevisiae* concentration was 2.14 × 10^6^ CFU/mL. After 30 s, the CFU/mL was reduced to 2.40 × 10^2^, and exposure for 45 s led to complete eradication. However, when the *S. cerevisiae* cells were exposed to plasma corona in wet mode, the nontreated *S. cerevisiae* concentration was 8.90 × 10^6^, and exposure for 120 s led to a reduction by five orders of magnitude; after prolonged exposure for 240 s, no CFU/mL was observed.

### 3.2. S. cerevisiae Viability as a Function of the Distance from the Sample to the Plasma Corona Discharge Treating Head

**Figure 3:**
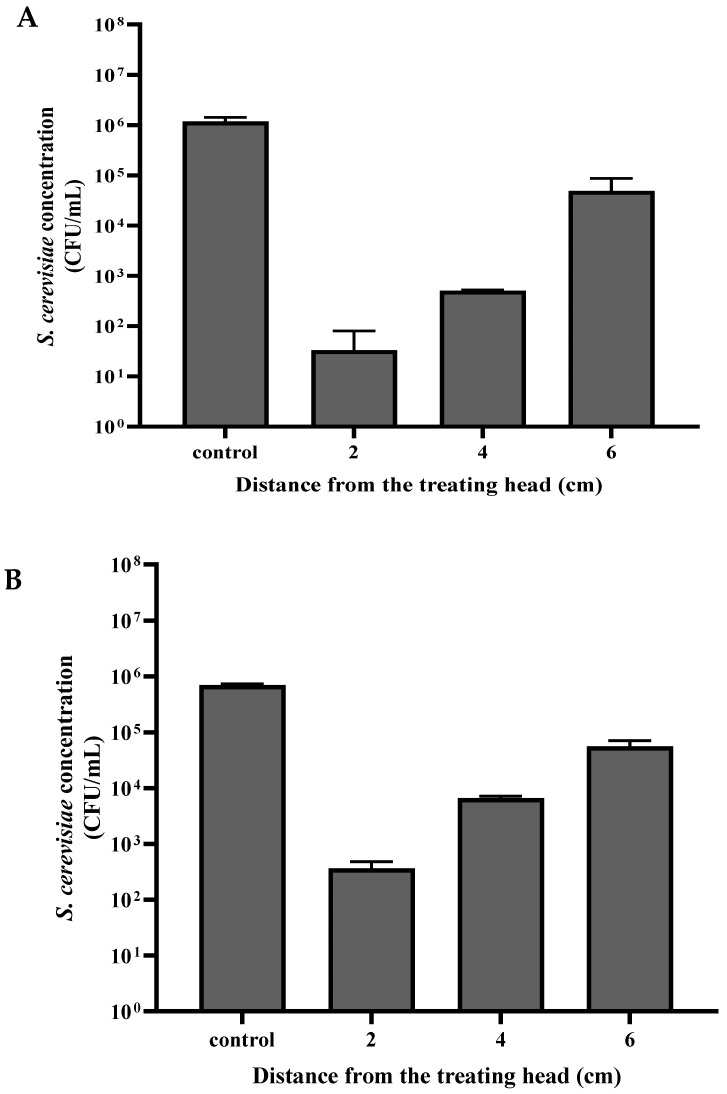
*S. cerevisiae* viability (CFU/mL) as a function of the distance between the sample and the plasma corona discharge treating head in dry mode, exposure for 30 s (**A**) and wet mode, exposure for 120 s (**B**), control column- without plasma exposure.

The *S. cerevisiae* sample in the Petri dish was placed at a distance of 2, 4, and 6 cm from the plasma treating head (Figure 3A,B). The dry-mode *S. cerevisiae* sample was exposed to plasma for 30 s, while the wet-mode sample was exposed for 120 s. The *S. cerevisiae* samples that served as controls were treated the same but without exposure to plasma treatment.

As can be seen in Figure 3, the distance of the sample from the treating head influenced the *S. cerevisiae* viability. In the control samples, the *S. cerevisiae* concentrations in dry mode and wet mode were 1.17 × 10^6^ and 6.93 × 10^5^ CFU/mL, respectively. The viability of cells that were exposed in dry mode at a distance of 6, 4, and 2 cm was 3.30 × 10^1^, 5.10 × 10^2^, and 4.92 × 10^4^ CFU/ mL, respectively. The viable cell concentration in wet mode decreased to 3.68 × 10^2^, 6.63 × 10^3^, and 5.60 × 10^4^ CFU/ mL, respectively.

### 3.3. S. cerevisiae Viability as a Function of the Initial Cell Concentration

A suspension of *S. cerevisiae* was grown overnight, washed, and diluted in PBS to ~10^7^, ~10^5^, and ~10^3^ CFU/mL. Each of the three suspensions was divided into volumes of 1 mL and centrifuged. For the dry-mode experiment, sediment (0.05 mL) from each concentration was dispersed in a Petri dish and exposed to plasma corona for 20 s. For the wet-mode examination, each concentration sample (0.05 mL) was diluted in 10 mL of PBS, placed in a Petri dish, and received 90 s exposure. The control samples were treated the same, except for the exposure to plasma.

**Figure 4:**
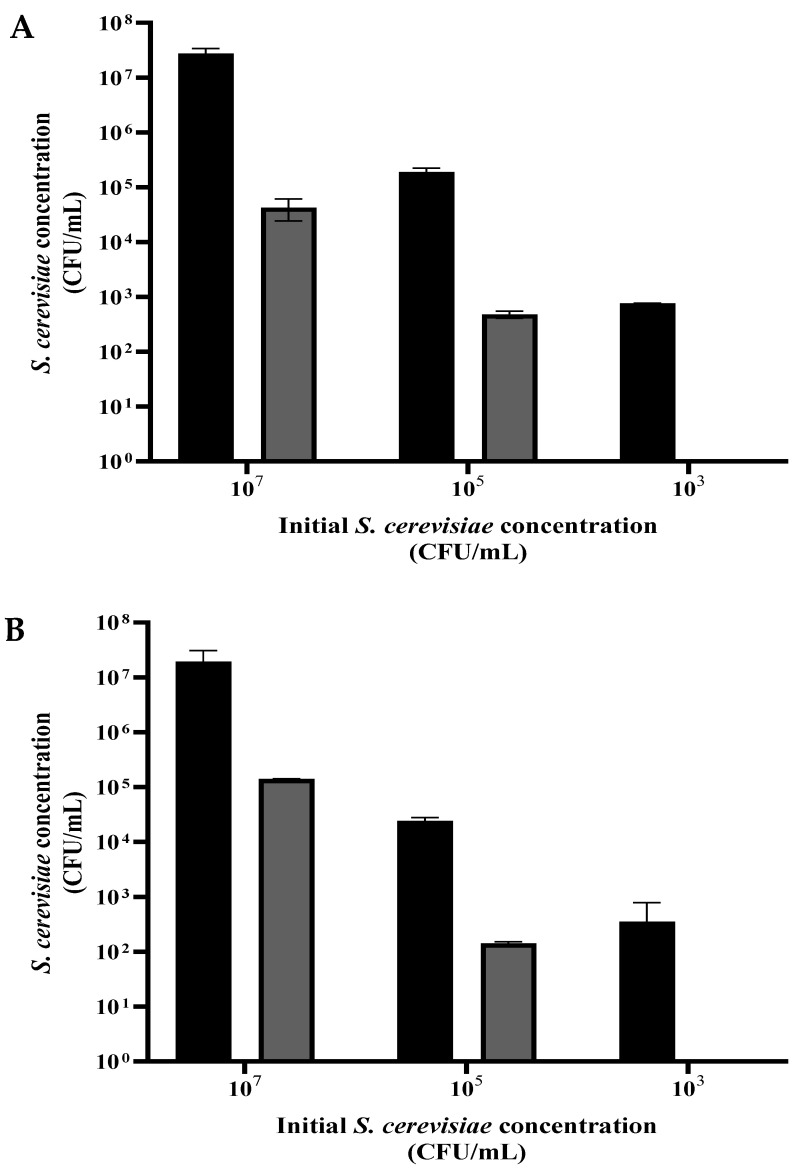
The concentration of *S. cerevisiae* (CFU/mL) as a function of the initial concentration, in dry mode, exposure for 20 s (**A**) and wet mode, exposure for 90 s (**B**). Grey and black column: with and without plasma treatment.

As shown in Figure 4A,B, exposure of the *S. cerevisiae* to plasma in dry (20 s) or wet (90 s) mode, with the different initial concentrations, resulted in a population reduction by 2 to 3 orders of magnitude. In both modes, there was total eradication when the initial cell concentration was about 10^3^ CFU/mL.

### 3.4. S. cerevisiae Viability as a Function of the Yeast Suspension Volume

A suspension of *S. cerevisiae* was grown overnight, then washed and diluted in PBS to 1.0 OD (~10^7^ CFU/mL). The suspension was divided into portions of 1 mL, centrifuged, and washed in PBS. The sediment was then diluted in 10, 20, and 30 mL. Each suspension was placed in a Petri dish. The liquid surface height in the Petri dish was 1, 2, and 3 mm, respectively. The samples were placed under the plasma treating head at a distance of 2 cm for 90 s. The control samples were treated the same, except for no exposure to plasma corona. This step was followed by a viable count assay was performed.

**Figure 5:**
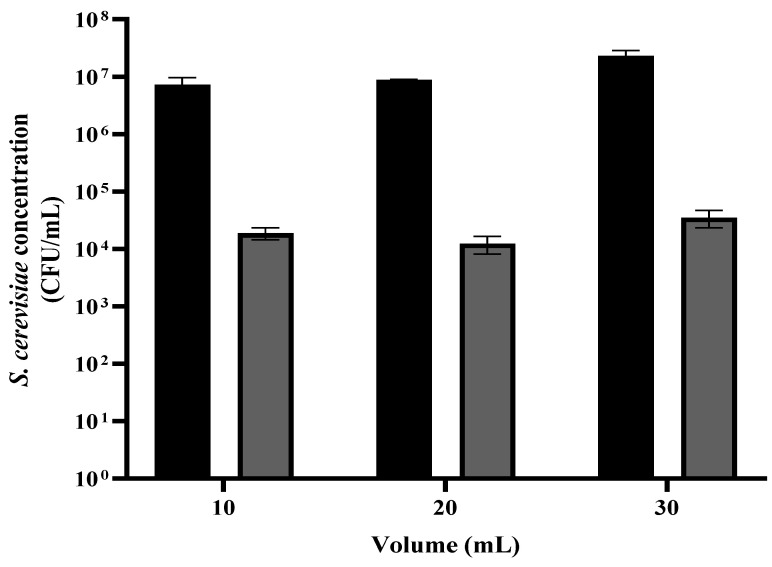
*S. cerevisiae* viability as a function of the yeast suspension volume. The samples were placed under the plasma treating head at a distance of 2 cm for 90 s. Grey and black column: with and without plasma treatment.

As shown in Figure 5, in all three initial volumes where the *S. cerevisiae* concentration was about the same (~10^7^ CFU/mL), the reduction in the yeast concentration was 2–3 orders of magnitude. The initial concentration of the control samples with 10, 20, and 30 mL were 7.32 × 10^6^, 8.83 × 10^6^, and 2.32 × 10^7^ CFU/mL, respectively. After the plasma treatment, the CFU/mL were 1.89 × 10^4^, 1.54 × 10^4^, and 3.51 × 10^4^, respectively. It may be important to note that the fan which connects the plasma corona facility with the rotating table led to high turbulence of the exposed liquid.

Pankaj et al. (2017) reported that treatment with nonthermal high-voltage (80 kV) atmospheric cold plasma for 4  min caused a 7.4 log CFU/ mL reduction in *S. cerevisiae* suspended in white grape juice, with no significant change in pH or electrical conductivity [31]. Gan et al. (2021) investigated cold plasma inactivation of *E. coli and S. cerevisiae* suspended in chokeberry juice (1–5 mL) after exposure for 1 to 5 min. When the treatment time was 4 min, the inactivation of *E. coli* and *S. cerevisiae* decreased as the sample volume increased. When a sample of 2 mL was treated for 4 min, the total number of *S. cerevisiae* and *E. coli* colonies declined by 1.31 and 2.83 log, respectively. Increasing the treatment time from 1 min to 5 min led to a higher eradication effect. When *E. coli* and *S. cerevisiae* were suspended in 2 mL with 4 min of plasma exposure, the colonies reduced by 2.27 and 1.23 log, respectively [32]. Stulič et al. (2019) examined the reduction of *S. cerevisiae* suspended in 190 mL 0.01 M NaNO_3_ sterile solution with a conductivity of 100 μS/cm. The samples were treated for 5 and 10 min. It was shown that treatment for 10 min led to a higher effect approaching total eradication (5.61 log10 CFU/mL) [33]. Wang et al. (2018) used plasma technology at different discharge voltages for inactivating yeasts (*Zygosaccharomyces rouxii* LB and 1130) in apple juice. When the initial populations of *Z. rouxii* LB and 1130 in apple juice were approximately 1 × 10^7^ CFU mL^−1^, plasma exposure at a peak discharge voltage of 11.3 kV for 30 min reduced their populations by 2.39 and 2.60 log_,_ respectively. By increasing the discharge voltages to 21.3 kV for the same treatment time, the number of *Z. rouxii* LB cells was reduced by 6.58 log, and that of *Z. rouxii* 1130 cells by 6.82 log [30]. Our results along with the cited results indicate a negative correlation between the sample volume and the corona plasma bactericidal effect. However, there is a linear correlation between treatment time and eradication effect.

### 3.5. Examination of S. cerevisiae Cell-Membrane Permeability

The *S. cerevisiae* cell membrane permeability was examined using flow cytometry analysis and the fluorescent dye propidium iodide (PI). When the cell membrane is damaged, PI enters the cell and binds to nucleic acid, displaying red under fluorescent light.

**Figure 6:**
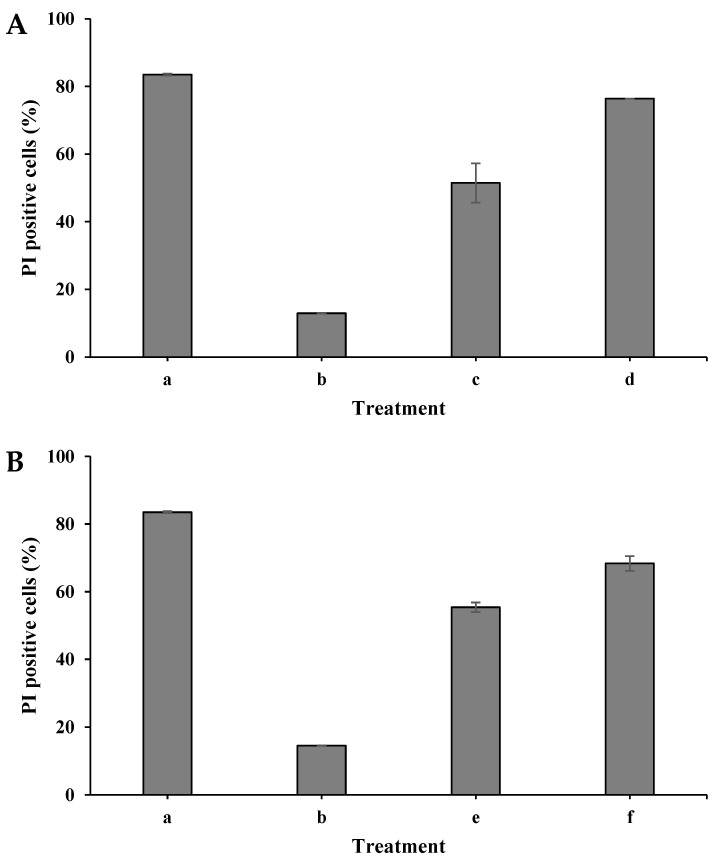

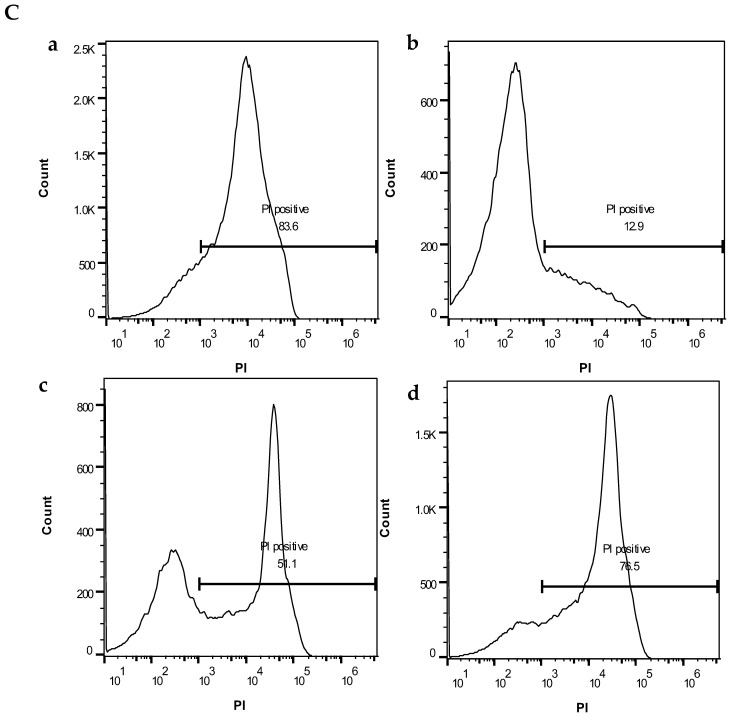
PI-positive *S. cerevisiae* as a function of plasma corona treatment in dry mode (**A**) and wet mode (**B**). *S. cerevisiae* cells that were treated with isopropanol (**a**); nontreated cells (**b**); *S. cerevisiae* cells that were exposed to plasma corona for 30 s (**c**); 45 s (**d**); 120 s (**e**); and 240 s (**f**). The histograms of the PI-positive cells in the dry mode (**a**–**d**) are shown in (**C**).

In these experiments, the cells were prepared for exposure to plasma in dry and wet modes, as described in Section 2.4, with an initial concentration of 10^6^ CFU/mL. The samples were placed at a distance of 2 cm from the treating head. When the cells were exposed to plasma in dry mode, the plasma corona exposure duration was for 30 and 45 s. After the treatment, the samples were collected from the plates into Eppendorf tubes using 1 mL PBS. When the cells were exposed in wet mode, the duration was for 120 and 240 s. Then, 1 mL of the 10 mL suspended cells in the Petri dish was collected. The PI dye was added to each sample and then incubated at 37 °C for 5 min. The samples were subjected to a flow cytometer, and the membrane permeability was examined. The same regime was performed for the control samples but without plasma exposure. A PI-positive control was prepared by incubating 10^6^ CFU/mL with isopropanol (100%) for 0.5 h. As shown in Figure 6A, the control sample, which was treated with isopropanol, exhibited 83% PI-positive cells. In contrast, the control of the nontreated samples exhibited only 13% and 15% in dry and wet modes, respectively. When the cells were plasma-treated in the dry mode for 30 s, the PI-positive cells were 51%. Longer exposure for 45 s increased the PI-positive cells to 76%. A similar result was observed when the *S. cerevisiae* cells were treated with plasma in the wet mode, Figure 6B. The histograms of the PI-positive cells in the dry mode (a–d) are shown in Figure 6C.

Xu et al. (2021) examined the cell viability of yeast using the dyes PI (staining dead cells or cells with damaged membranes) and SYTO 9 (staining cells with intact membranes). The survival rate significantly reduced in a time-dependent manner, observed as 81.3%, 34.6%, and 19.0% after 2.5, 5, and 10 min plasma treatments, respectively [34]. Gan et al. (2021) showed flow cytometry results from plasma-treated *S. cerevisiae* and *E. coli*, where the percentage of green-colored cells gradually increased when the cells were plasma-treated (suspended in 2 mL, treatment time of 1–5 min). The percentage of green-colored *E.coli* cells rose from 4.2% to 19.0%, which was correlated with longer treatment time. Similar observation was reported for *S. cerevisiae,* where the green-colored cells percentage was increased from 4.8% to 10.7% with a treatment time of 1–5 min [32].

### 3.6. Examination S. cerevisiae Morphology

**Figure 7:**
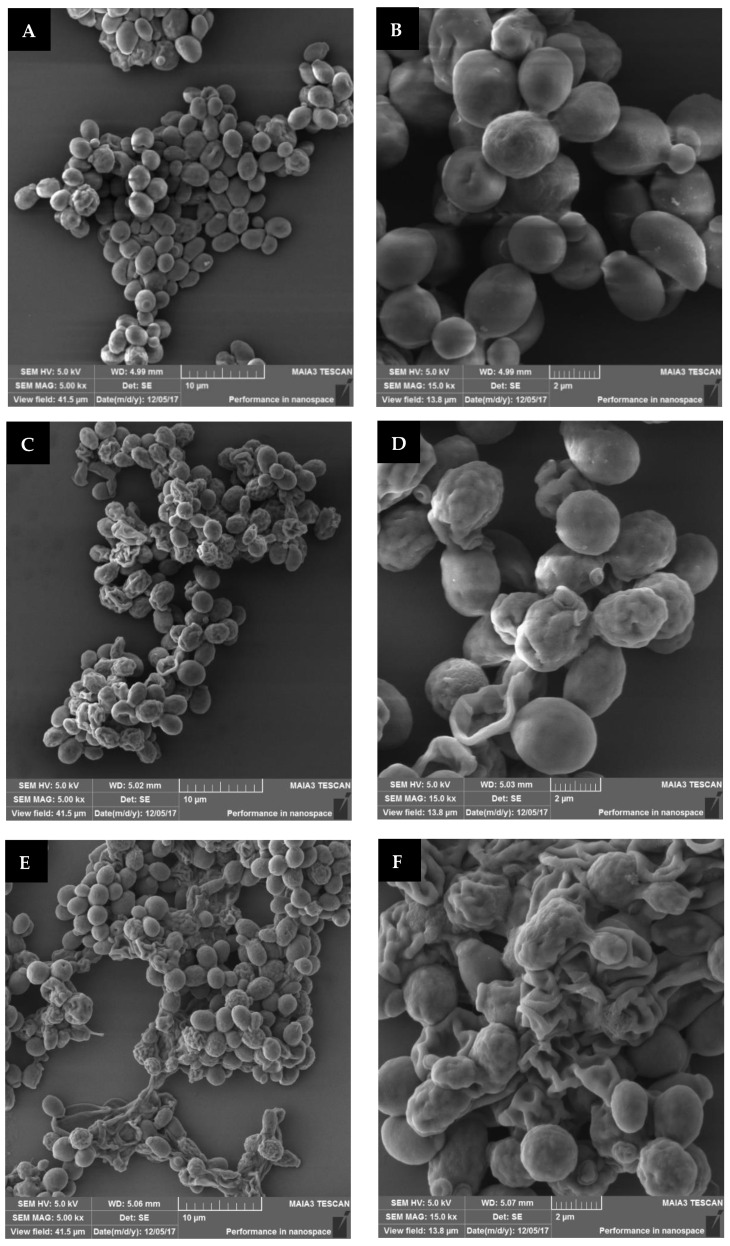
SEM images of nontreated *S. cerevisiae* cells (control) (**A**,**B**); plasma-treated cells for 30 s (**C**,**D**); and 60 s (**E**,**F**). Magnification of 5 kx (5000 fold) (**A**,**C**,**E**) and 15 kx (15,000 fold) (**B**,**D**,**F**).

The *S. cerevisiae* cells were prepared for plasma treatment in dry mode. Sediment (10^6^ CFU/mL) was spread on a Petri dish, placed under the treating head at a distance of 2 cm, and exposed to plasma corona for 30 and 60 s. Samples that were prepared the same way but without exposure were used as controls. The samples were collected from the plate and fixed for SEM analysis. The images of the plasma-treated and control samples at a magnification of 5 kx and 15 kx are shown in Figure 7.

In Figure 7A,B, it can be seen that the nontreated *S. cerevisiae* samples mainly included intact cells, some of which were in the budding process. The plasma-treated images Figure 7C–F, show deformed cells. Treatment for 60 s led to about 84% of defective cells (E,F), compared to 30 s of treatment, which led to 71% (C,D). The nontreated sample, in contrast, exhibited only 13% of defective cells, probably due to the fixation procedure for SEM analysis (A,B).

Gan et al. (2021) studied the effects of a cold plasma jet with a dielectric barrier configuration on *E. coli* and *S. cerevisiae* suspended in chokeberry juice. SEM images showed that the nontreated *E. coli* and *S. cerevisiae* cells were oval in shape, with smooth and intact surfaces. However, plasma-treated *E. coli* cells exhibited noticeable damage, including breakages, folds, and dents; and plasma-treated *S. cerevisiae* cells were typically stretched and slightly broken with some surface dents [32]. Stulič et al. (2019) analyzed *S. cerevisiae* morphology using transmission electron microscopy (TEM). Their images showed nontreated yeast cells exhibiting well-defined cellular organelles (nucleus, mitochondria, and endoplasmic reticulum). After plasma treatment, disintegrated cells were observed, with some completely vacuolated, so their organelles and cell membranes were indistinguishable. In addition, there was partial leakage of cellular contents into the surrounding medium [33]. Wang et al. (2018) reported that plasma-treated yeasts showed severe damage, with rough, ridged surfaces containing discrete holes [30]. Xu et al. (2021) analyzed ROS generation (^·^OH, ^1^O_2_, **^·^**O_2_^−^, and H_2_O_2_) in a plasma-liquid interaction system. They suggested that plasma can effectively inactivate yeast cells mainly by destroying their membranes, attributed to ^·^OH and H_2_O_2_. In contrast, cell metabolism disruption was attributed primarily to ^1^O_2_. SEM images revealed that plasma treatments wrinkled and disrupted yeast cells, producing cell debris. It was reported that the degree of damage increased together with plasma treatment time [34].

The results of our study showed a linear correlation between the exposure duration and the reduction of the CFU/mL in both dry and wet modes. The eradication effect of the plasma treatment was higher when the sample was treated in the dry mode. The distance between the sample and the treating head was an essential factor, with shorter distance increasing the efficiency of eradication. Another critical factor was the *S. cerevisiae* initial concentration. In both modes, there was total eradication when the initial cell concentration was about 10^3^ CFU/mL. When different volumes with the same *S. cerevisiae* concentration were exposed to plasma while mixing the suspensions, the same decrease, about 3 orders of magnitude, was observed. From these results, we assume that this technology may be applied on a large scale. However, the plasma exposure must be performed continuously, including the time when the liquid is agitated while passing through a narrow pipe.

Examination of the cell-membrane permeability using a flow cytometer showed that plasma treatment led to penetration of the PI fluorescent dye into the cells. SEM analysis confirmed that plasma treatment led to cell deformation. We assume that, since plasma treatment caused physical damage, such as holes in the cell membrane and cell deformation, this technology may be useful for the eradication of different microorganisms.

## 4. Conclusions

*S. cerevisiae* is a major contaminating microorganism associated with fruit juices. The inactivation of microorganisms in the food industry is commonly performed with thermal treatments. However, this treatment can affect the nutritional properties. Several studies including our study emphasized microorganism eradication using cold plasma technology.

In this study, the effect of cold plasma exposure on *S. cerevisiae* viability was examined when the cells were prepared in two modes: suspended in PBS (wet mode) and concentrated in a minimum volume of PBS (50 µL) (dry mode). The results showed a linear correlation between the exposure duration and the eradication effect. When the initial concentration was about 10^6^ CFL/mL, complete eradication was observed after exposure for 45 and 240 s in the dry and wet modes, respectively. The distance between the sample and the plasma treating head (6, 4, and 2 cm) influenced the *S. cerevisiae* viability. When the plasma treating head was adjusted at 2 cm from the sample, the highest eradication (two orders of magnitude) was observed after treating times of 30 s for the dry mode and 90 s for the wet mode. When different initial cell concentrations were examined, a total *S. cerevisiae* eradication was observed when the initial cell concentration was about 10^3^ CFU/mL. Plasma treatment (90 s) of different volumes while mixing the suspended *S. cerevisiae* showed a reduction of two orders of magnitude. In addition, plasma exposure led to PI permeability, and SEM analysis showed cell deformation. 

We assume that, since plasma treatment caused physical damage, such as holes in the cell membrane and cell deformation, this technology may be useful for eradicating different microorganisms. Applying this technology on a large scale should be done when the liquid is agitated continuously. The main advantage of cold plasma technology for microorganism eradication in the fruit juice industry is that disinfection is achieved in a short process without heating.

## Data Availability

Data are available in this publication.
